# Breastfeeding and lactation research: exploring a tool to measure infant feeding patterns

**DOI:** 10.1186/1746-4358-9-5

**Published:** 2014-04-24

**Authors:** Joy Noel-Weiss, Monica Taljaard, Sonya Kujawa-Myles

**Affiliations:** 1 School of Nursing, University of Ottawa, 451 Smyth RGN3051, K1H 8 M5, Ottawa, ON, Canada; 2 Department of Epidemiology and Community Medicine, Clinical Epidemiology Program, Ottawa Hospital Research Institute, University of Ottawa, 1053 Carling Avenue, K1Y 4E9 Ottawa, ON, Canada

## Abstract

**Background:**

Infant feeding categories, often referred to as breastfeeding definitions, form the basis to describe infant feeding patterns; especially, breastfeeding duration and degree of breastfeeding exclusivity. Researchers use a variety of algorithms and no validated tool exists to measure feeding patterns for research purposes. The goal of this research project was to develop and test a tool to measure patterns of infant feeding for breastfeeding and lactation research.

**Methods:**

We used a literature review, survey methods, and statistical analysis to develop and test this health measurement tool. The work was completed in three phases: 1) development of the tool; 2) assessment of content validity with a panel of experts; and 3) testing for inter-rater reliability by comparing the conclusions of 2 independent research assistants (RAs) and by comparing mothers’ feeding diaries with the RAs’ findings. For the third phase, an a priori analysis determined we needed to recruit 75 participants. Inclusion criteria were women who had given birth to a single healthy newborn, planned to breastfeed and were able to breastfeed freely, were able to read and write in English or French, were willing and able to maintain a weekly feeding diary for 6 weeks and to answer 6 English telephone questionnaires (twice within 24 hrs x 3 times over 6 months. To measure inter-rater reliability, we used intraclass correlation coefficient.

**Results:**

The final tool, The FeedCat Tool, contains two parts: 1) questions asked to determine what and how the baby was fed and 2) a chart to indicate the feeding category for each time point and recall period. We recruited 75 breastfeeding mothers to measure inter-rater reliability. Inter-rater reliability for classification of feeding categories by the two RAs and for agreement between the RAs’ findings at 1 month and mothers’ diaries at 4 weeks indicated excellent agreement.

**Conclusion:**

We produced a feeding categories tool that can be used by researchers to describe the type, amount, and mode of feeding, and we tested the tool for content validity and reliability. Researchers should consider The FeedCat Tool for lactation and research projects requiring data about infant feeding patterns.

## Background

For more than two decades, researchers have sought consistency in definitions for breastfeeding, and they used various algorithms to determine how much a baby is breastfed [1,2]. These definitions formed the basis to describe infant feeding patterns, especially, breastfeeding duration and the degree of breastfeeding exclusivity. However, no validated tool exists to identify feeding categories for research purposes. In the absence of a tool tested for reliability, a researcher cannot be sure that if two different research assistants called the same study participant, on the same day, they would get the same answer.

Along with testing for reliability, an algorithm or tool ought to be tested for validity. A researcher needs to know that a tool measures what it is intended to measure. Recently, the authors questioned the terminology used to describe breastfeeding, specifically, that descriptions should incorporate the method of feeding in addition to how much breast milk the baby receives [3]. As well, many researchers do not report the definitions and descriptors they used for their work [4]. This lack of breadth and clarity is a problem, since two of the goals of consistent definitions are to ensure comparability of research studies and generalizability of findings.

The purpose of this research project was to develop and test a tool to measure patterns of infant feeding for breastfeeding and lactation research. The goals were to produce a feeding categories tool for researchers to use that would include type, amount, and mode of feeding and to check the tool for reliability.

## Methods

We used a literature review, survey methods, and statistical analysis to develop and test this health measurement tool. The study was designed based on Streiner and Norman’s book, *Health Measurement Scales: A Practical Guide to their Development and Use*[5]. In particular, we used their definitions and directions regarding reliability and validity, since our primary intent was to check for consistency and accuracy. The work was completed in three phases: 1) development of the tool; 2) assessment of content validity; and 3) testing for inter-rater reliability.

### Phase I

Initial drafts of the tool were created based on a methodical review of the literature to find definitions used for infant feeding categories and on the authors’ clinical and research experience. It was designed for use by researchers or research assistants who will ask study participants questions and then complete the chart of feeding categories and scores. We attempted to provide flexibility with respect to timing of data collection and to account for quantity of breast milk and mode of feeding in the first six months following birth (i.e., during the typical period of exclusive breastfeeding). To capture breastfeeding patterns over time, we added a scoring system, so multiple time points could be averaged for a single score. The tool does not collect data about the introduction of complementary foods, specifically, the soft, semi-solid, and solid foods introduced around the middle of the first year.

### Phase II

To assess the face and content validity of the initial tool, we consulted clinical and research experts in the field of breastfeeding and lactation to review the draft (see Additional file 1). We asked for feedback about the tool’s content, specifically, whether they believed we captured descriptions of breastfeeding patterns that would be useful for research projects. The revised tool (see Additional file 2) was then pilot tested with research assistants to determine readability, usability, and burden for potential users.

### Phase III

For the third phase, breastfeeding women were recruited then telephoned by research assistants who worked in pairs. Breastfeeding mothers were recruited following the birth of their baby and before discharge from the postpartum unit of a large Canadian hospital that averages 6,200 births per year [6]. Inclusion criteria for the study were women who had given birth to a single, healthy newborn, planned to breastfeed and were able to breastfeed freely, were able to read and write in English or French, were willing and able to maintain a weekly feeding diary for 6 weeks and to answer 6 English telephone questionnaires (twice within 24 hrs × 3 times over 6 months). Nurses asked patients who met the criteria if they would be willing to learn about the study, then the researcher or a research assistant explained the study to interested mothers and obtained a signed consent and a questionnaire of demographic information.

Research assistants (RAs) collected data at 1 month, 3 months, and 5 months following recruitment. One RA was a registered nurse and certified lactation consultant, one RA was a fourth year nursing student who had completed a maternity course, and one RA was a third year nursing student who had not completed a maternity course. The rationale for selection of RAs was to avoid bias due to expertise in the field of maternity and breastfeeding care. The three RAs were randomized into pairs to make the telephone calls, then the two RAs called study participants within 48 hours of each other to administer the tool. After a first call was completed, the first caller would text her partner to inform her that the second call could be made.

To further test validity (i.e., that we were measuring what we intend to measure), we compared mothers’ diaries with the categories RAs recorded. Participants were given a feeding diary with eight boxes to tick each week for six weeks (see Additional file 3). The fourth week of the diary was compared to the 1 month telephone call made by the first caller. Without knowing the category recorded by the RA, the researcher determined a feeding category based on the boxes ticked by the mother. The two categories were then compared for levels of agreement.

### Data analysis

Characteristics of mothers and newborns were summarized using descriptive statistics (mean and standard deviation for continuous variables and frequencies and proportions for categorical variables). Agreement between the two callers and agreement between diaries’ and RAs’ classification of feeding categories was assessed using an intraclass correlation coefficient (ICC). For the ICC, we used two-way mixed and absolute agreement options.

While the score using this tool is correctly an ordinal (categorical) score, it has been suggested that kappa does not function as well beyond the 2 by 2 table and it does not take into account the distance between scores on an ordinal scale [7,8]. Weighted kappa’s can be used for data of this type but a number of authors have identified problems inherent in its use, and Maclure and Willett concluded that “a logical choice of standard weights makes weighted kappa equivalent to the intraclass correlation coefficient” [7,8]. For these reasons, the data was treated as ordinal-continuous and the intraclass correlation coefficient (ICC) in SPSS 20 was used to calculate the inter-rater reliability as well as agreement between the RA’s rating and the mother’s diary.

### Sample size calculation for Phase III

While some researchers have based sample size calculations for ICCs on tests of hypotheses, others have argued that it makes more sense to base sample size calculations on attaining a specified level of precision around the ICC [9]. We anticipated there would be high levels of agreement (0.8 or more) between raters and between the tool and criterion (diary). Based on this supposition, assuming an alpha of 0.05 and a desired width of confidence interval of 0.2, we estimated that 55 subjects would be required [10]. In order to account for 30% attrition due to rates of early weaning and loss to follow-up over the 6 month data collection period, 75 subjects were recruited.

### Research ethics

The University of Ottawa Research Ethics Board [File Number: H11-11-02] and the Ottawa Hospital Research Ethics Board [Protocol # 20120200-01H] approved the study. The study was conducted in English but all recruitment material was also available in French.

## Results

### Phase I - developing the tool

The final tool, labelled FeedCat Tool, is presented in Additional file 4. It contains two parts: 1) questions asked to determine what and how the baby was fed; and 2) a chart to indicate the feeding category for each time point and recall period. The Feedcat Tool used for the data collection (see Additional file 2) included a scoring system because we were trying to capture breastfeeding patterns by assigning a score to the types of feeding and amount of breast milk. The scoring system provided an average of the scores over time, but this number was not useful when we completed our analysis.

The form was designed to be flexible regarding time points and recall periods for data collection. The number of data collection intervals are optional, and each data collection interval should specify time point and recall period (e.g., at 1 month/24 hour recall, at 5 months/7 day recall). Time points should be determined by the research purpose and the research question, as it could be necessary to track patterns over time or at set time points. For example, if the research question is about maternal self-efficacy, then the researcher will probably be collecting data at fixed time points to determine how mothers are feeding at that time. On the other hand, the priority might be how the baby is fed from birth. In that case, the questionnaire would be asked more frequently to ensure accuracy, and it would pick up from where the last questionnaire finished to provide a complete pattern.

### Phase II - assessing content validity

Eleven experts were asked for feedback about the tool. All experts had worked with breastfeeding mothers and babies, and eight experts were also researchers with published papers. The nine experts who responded represented Australia, Canada, Sweden, the United Kingdom, and the United States. There were recommendations to clarify language, to simplify the administration, and to reformat the chart. Response to the use of scores was mixed; some liked it, some did not, and others did not comment.

### Phase III - testing reliability

Seventy-five breastfeeding mothers were recruited for the study from the postpartum unit during three weeks in July and August 2012 (see Table 1 for participant characteristics). We did not record how many patients were approached by the nurses, but very few refused after the researcher or a research assistant explained the study. The main reason given for not joining the study was “too busy”.

**Table 1:** Characteristics of participating mothers and newborns

***Characteristics***	***Frequency*** **(%)**	***n***
Maternal age (years) [*+/−*SD(range)]	33*+/−*5.6 (17–43)	72
In committed relationship (%)	69 (97)	71
Completed post-secondary education (%)	61 (87)	71
Family income > $70,000 (CAN) (%)	49 (65)	68
First spoken language (%)		72
English	45 (63)	
French	15 (21)	
Other	12 (17)	
Primiparous women (%)	34 (47)	73
How long plan to breastfeed without other foods (%)		71
Less than 6 months	5 (7)	
6 months	55 (78)	
Not sure	11 (16)	
How long plan to breastfeed overall (%)		71
6 months or less	7 (10)	
7 - 12 months	8 (11)	
12 months	35 (49)	
More than 1 year	11 (16)	
Not sure	10 (14)	

Ideally, to measure inter-rater reliability, we wanted the calls to be as close together as possible, but several factors made it difficult including availability of the second RA and availability of mother (e.g., if a participant left to shop or go on vacation after the first call or the second RA was in class or at work). We included all calls made within 48 hours of each other, even though the recall for one time point was past 24 hours. The second caller asked if there had been any significant change in the past 24 hours (e.g., separation between mother and baby or the start of supplements) but the majority of participants said no. These responses were verified, as evidenced by the high inter-rater reliability, and showed that mothers were staying consistent from one day to the next.

Thirty-six participants finished two-paired calls at all three time points. Of the possible 225 paired calls (i.e., 75 participants with 3 collection times), 143 two-RA calls were completed within 48 hours, 17 paired calls were removed because they were more than 48 hours apart, and 9 lacked data because the second caller could not reach the participant (see Table 2 for a description of participation).

**Table 2:** Participation characteristics

***Characteristics***	***Result***
Number recruited	75
Number to complete at least one session	65
Number lost to follow-up	10
Number weaned (i.e., cessation of all breastfeeding)	14
Number of 2-call sessions completed within 48 hours (% of 143 completed calls)	143
1 month	55 (39%)
3 months	49 (34%)
5 months	39 (27%)
Number of 2-call sessions invalidated because second call was more than 48 hours after first call	17
Number of sessions with no second call	9
Number of sessions missed due to prior weaning	21
Number of 1-call sessions with no follow-up due to weaning (i.e. mothers upset about weaning)	5

Initially, the protocol included calling women who had weaned (i.e., stopped all breastfeeding) again at the next data collection time to establish whether or not they had restarted breastfeeding. In reality, this strategy turned out to be stressful for women, and we stopped calling if a woman said she had stopped breastfeeding. In 5 cases, when the first caller learned a woman had weaned her baby and sensed she might be distressed, the second call was not done out of respect for the mother’s feelings.

Inter-rater reliability for classification of feeding categories by the two RAs indicated excellent agreement (see Table 3). The intraclass correlation coefficient (ICC) measuring agreement between the two RAs ranged from 0.90 (95% CI 0.826 to 0.941) to 1.00 (95% CI 0.996 to 0.999) across the various time points. Agreement between the RAs feeding category at 1 month and mothers’ diaries at four weeks was measured as 0.80 (95% CI 0.662, 0.884; see Table 4). An ICC above 0.75 is considered excellent [11].

**Table 3:** Inter-rater reliability (ICCs) for feeding categories at 1, 3, and 5 months with 24-hour and 7-days recalls

***Comparison***	***n***	***ICC***	***p-value***	***95% Confidence interval***
All calls				
Past 24 hours	143	0.958	<0.001	(0.941, 0.970)
Past 7 days	143	0.974	<0.001	(0.963, 0.981)
One month call				
Past 24 hours	55	0.898	<0.001	(0.826, 0.941)
Past 7 days	55	0.937	<0.001	(0.893, 0.963)
Three month call				
Past 24 hours	49	0.997	<0.001	(0.996, 0.999)
Past 7 days	49	0.996	<0.001	(0.993, 0.998)
Five month call				
Past 24 hours	39	0.984	<0.001	(0.969, 0.991)
Past 7 days	39	0.990	<0.001	(0.982, 0.995)

**Table 4:** Prevalence of breastfeeding categories from mothers’ diaries and agreement between diaries and RA rating at one month

***Characteristics***	***Frequency*** **(%)**	***n***
Number of 6-week diaries completed		30
Feeding categories at 4 weeks	16 (54)	30
Exclusively breastfed	7 (24)	
Exclusively breast milk-fed	6 (20)	
Predominately breast milk-fed Weaned (i.e., stopped all breastfeeding)	1 (1)	
Mother’s diary at 4 weeks agreed with 1 month call (7-day recall) by 1^st^ and 2^nd^ callers		55
Yes	46 (84)	
No	9 (16)	
[5 of 60 missing]		
*Reliability test comparing callers with diaries*	*Measure*	*p-value*
ICC	0.802	(*p* < 0.001)
	(95% CI 0.662, 0.884)	

Table 5 presents the observed prevalence in our sample within each of the infant feeding categories over time. This homogeneous, self-selected group of participants was clustered in four categories with five of the nine categories having either none or only one mother at each of the six data collection intervals. The majority of mothers were exclusively breastfeeding, and the comparison of 24-hour recall with 7-day recall demonstrates uneven patterns of feeding with women moving in and out of categories over time. The higher rate on the 24-hour recall than on the 7-day recall at every data collection interval indicates mothers would use a supplement occasionally over the week. The sample size fluctuated with the data collection intervals, as some mothers who were not reached at one interval may have completed one or two of the other intervals (e.g., RAs might not have reached a mother at 1 month, but she completed the sessions at 3 months and 5 months).

**Table 5:** Description of infant feeding categories reported in completed sessions to first caller (n = 75)

	***Frequency (%)***					
	**1-month**		**3-month**		**5-month**	
	***n*** **= 63**		***n*** **= 53**		***n*** **= 47**	
***Characteristics***	**24 hour recall**	**7 day recall**	**24 hour recall**	**7 day recall**	**24 hour recall**	**7 day recall**
1) Exclusively breastfed			38 (51)	27 (36)	31 (41)	25 (33)
2) Exclusively breast milk-fed	42 (56)	31 (41)	6 (8)	14 (19)	5 (7)	7 (9.3)
3) Predominately breastfed	5 (7)	12 (16)	0	0	0	1 (1)
4) Predominately breast milk-fed	1 (1)	1 (1)	3 (4)	6 (8)	5 (7)	8 (11)
	8 (11)	12 (16)	0	0	0	0
5) Partially breastfed	0	0	0	0	1 (1)	1 (1)
6) Partially breast milk-fed	0	0	0	0	0	0
7) Minimally breastfed	0	0	0	0	0	0
8) Minimally breast milk-fed	1 (1)	1 (1)	6 (8)	6 (8)	5 (7)	5 (7)
9) Weaned (no breastfeeding)	6 (8)	6 (8)	22 (29)	22 (29)	28 (37)	28 (37)
Missing	12 (16)	12 (16)				

## Discussion

This work was complex. To account for modes of feeding, we debated wording to differentiate between babies fed at their mothers’ breasts and babies fed breast milk by other means. In the end, we chose “breastfed” and “breast milk-fed” as the terms. We also describe feeding from the baby’s point of view (i.e., breastfed) as opposed to most definitions which use “breastfeeding”. These terms were coupled with typical quantitative terms: exclusively, predominately, partially, and minimally.

The experts’ evaluations were an essential step in developing the tool. Their feedback helped build the tool and develop the procedure for identifying feeding patterns. Having a mix of clinicians and researchers provided a variety of suggestions.

For our purposes, we used two columns for each time point (i.e., 1 month, 3 months, 5 months) and asked for a 24-hour recall and a 7-day recall. After the 3-month set of calls, the RAs met with the researcher and adjusted the list of questions. With experience, they found easier ways to ask questions; discovered asking about soothers and who was feeding the baby were pointless questions offering little insight into the feeding categories, whereas, asking about pumping and what mothers did with pumped milk was a useful question; and they needed some guidance for how much a feeding supplement was used for replacement at each age (e.g., 5 month old babies take about 100 mls. per feed so a top up of 25 mls. would be about a 25% replacement).

The rationale for offering a scoring option is that time point checks do not capture the over-time pattern. Mothers do not consistently transition from exclusive breastfeeding to partial breastfeeding and, finally weaning [12]. For example, a woman might supplement her baby once or twice in the first few days but then breastfeed exclusively until six months or an infant might have a nursing strike (refusal to breastfeed suddenly) but, with coaxing and patience, the strike is overcome and the dyad carries on with exclusive breastfeeding. In the end, the potential for the scoring system was not explored with this study. While the idea might have merit, it would need to be tested, and the final version of the FeedCat Tool does not use the scoring system.

Our goals were to produce a feeding categories tool that could be used by researchers to describe the type, amount, and mode of feeding and to validate the tool for content validity and reliability. We demonstrated high agreement between two RAs administering the tool independently within a 48 hour period. Coupled with a substantial agreement between RAs and mothers’ diaries, our tool is both reliable and valid.

The limitations in this work involve the small, homogeneous sample. Regarding the expanded terms for types of feeding, the terms were not really tested since few babies fit under these categories. The scoring system provided by the chart was also not tested, since this validation study did not have a research question that required such a score. Additional research is needed with a larger sample to further test the categories and the scoring option.

## Conclusion

Researchers need internationally accepted definitions of infant feeding patterns for breastfeeding and lactation research. These descriptions should be captured in the form of a validated tool. This study explored a new method to capture breastfeeding, and it incorporated how the baby was fed along with what the baby was fed, as well as offering the option of collecting data at specific time points or as a lifelong pattern. We have shown high agreement between pairs of RAs who have collected the data with this sample. The FeedCat Tool should be considered for data collection.

## Competing interests

JN-W received an internal grant from the University of Ottawa to complete this study. The authors declare that they have no competing interests.

## Authors’ contributions

JN-W identified the need for a validated tool and conceived the first version. All authors contributed to the research design. JN-W managed the research project, completed the data analyses, and wrote the first draft. All authors contributed to interpretation of the results and to the pre-submission revisions. All authors approved the final manuscript.

## Supplementary Material

Additional file 1 Prototype Infant feeding category assessment tool (FeedCat Tool). Click here for file

Additional file 2 Infant feeding category assessment tool with scoring used for study. Click here for file

Additional file 3 Mother’s diary in pamphlet format. Click here for file

Additional file 4 Infant feeding category assessment tool (FeedCat Tool). Click here for file
