# Supplementary material for: Breastfeeding and lactation research: exploring a tool to measure infant feeding patterns
Source: Int Breastfeed J. 2014 Apr 24;9:5. doi: 10.1186/1746-4358-9-5 (PMC4022273; doi:10.1186/1746-4358-9-5)
Supplement: Additional file 1 — Prototype Infant feeding category assessment tool (FeedCat Tool). [file 1746-4358-9-5-S1.doc]

**Additional file 1 - Prototype Infant feeding category assessment tool (FeedCat Tool)**

Infant Feeding Category Assessment Tool

Part I - Open-ended questions/statements to gain an overall picture.

Researcher to mom - "I will ask questions in two parts to try to get a clear picture of how you have been feeding your baby. First, I have six general questions."

1. How has feeding been going? Rationale - start by asking a general question
2. What has your baby been eating? Rationale - ask about food - all breast milk? - to get a general idea of what baby eats. Medicines and vitamins do not count as alternates to breast milk, but water and tea does substitute for breast milk.
3. Besides nursing your baby at your breast, please describe other ways you have used to feed your baby. Rationale - ask about method - all at mom's breast? - again, to get a general idea.
4. Who usually feeds your baby? Rationale - also asks about method - all at mom's breast? - may find mom remembers differently when asked differently.
5. How often in 24 hours does your baby usually eat? Rationale - helps to prompt mom to think about feeding patterns and may help in Part II with determining average number of feeds.
6. What types of equipment have you used? Rationale - to get a general picture of pumps, laction aids, and bottles.

Part II - Closed questions to complete a score.

Researcher to mom - "I will now ask several yes/no questions to get a specific pattern for how you have fed your baby."

Researcher should ask 1) **the initial statement** (e.g., "In the past 24 hours," or "In the past 7 days," or "In hospital,"); 2) **with** **the question** (e.g., "Did your baby receive only breast milk?") to determine pattern of infant feeding (note the clarifications in the parentheses); then **follow up with the next indicated question** (e.g., "Yes to all – Question 2; No to any – Question 3") until a Yes (Y) is reached. Recommend you work through one column at a time.

ONLY one Yes (Y) should be circled in each column. Add all columns and divided by the number of columns (i.e., in this case add the four numbers and divide by 4) to get a score out of 10.

| Category  Question (clarification)  Category/Next question | Initial statement | In the past 24 hours, | In the past  7 days, | In hospital, | Since  your baby's birth, |
| --- | --- | --- | --- | --- | --- |
| Exclusive breast milk - first question, not a category  Has your baby had anything to eat or drink besides breast milk? (vitamins and medication not included) **No, he/she had only breast milk – Go to Question 1;**  **Yes, he/she had something besides breast milk** (e,g, solids, formula, water, tea) **– go to Q3; No breast milk at all - go to Q9** | |  |  |  |  |
| 1) Exclusively breastfed  Has your baby received all his/her feedings/nursings at your breast?  Yes – no further questions; **No, I used bottle, finger, cup, other – go to Q2** | | Y = 10  N | Y = 10  N | Y = 10  N | Y = 10  N |
| 2) Exclusively breast milk-fed  Your baby received only breast milk and you used bottle, finger, cup, other?  Yes – no further questions | | Y = 8 | Y = 8 | Y = 8 | Y = 8 |
| 3) Predominately breastfed  Did your baby receive mostly breast milk (75% or more) and were all supplements were at your breast? (3/4 breast milk or more - all nursing at mom's breast; i.e., used formula with tube at breast and no bottle, finger, cup, other)  Yes to both - no further questions; **It was 75% but I used bottle, finger, cup, other - go to Q4**; **No, it was not 75% or more** - **ask mom to specify half or quarter was breast milk: if 1/2 were breastmilk - go to Q5 OR if 1/4 or less were breastmilk - go to Q7** | | Y = 8  N | Y = 8  N | Y = 8  N | Y = 8  N |
| 4) Predominately breast milk-fed  Your baby received mostly breast milk and you used bottle, finger, cup, other?  (3 of 4 or more feeds were breast milk; some bottle, finger, or cup)  Yes – no further questions | | Y = 6  N | Y = 6  N | Y = 6  N | Y = 6  N |
| 5) Partially breastfed  Did your baby receive about half breast milk, and all nursing was at your breast?  (half of food was breast milk, all nursing at mom's breast; i.e., used formula with tube at breast and no bottle, finger, or cup)  Yes – no further questions; **No, I used bottle, finger, cup, other – Question 7** | | Y = 6  N | Y = 6  N | Y = 6  N | Y = 6  N |
| 6) Partially breast milk-fed  Your baby received about half breast milk with some feeds by bottle, cup or other ways? (2 of 4 feeds daily were breast milk, used bottle, finger, or cup)  Yes – no further questions | | Y = 4 | Y = 4 | Y = 4 | Y = 4 |
| 7) Minimally breastfed  Did your baby receive some (25% or less) breast milk, and all nursing was at your breast? (1 of 4 feeds or less daily were breast milk, all nursing at mom's breast; i.e., used formula with tube at breast and no bottle, finger, or cup)  Yes – no further questions; **No, I used bottle, finger, cup, other – Question 8** | | Y = 4  N | Y = 4  N | Y = 4  N | Y = 4  N |
| 8) Minimally breast milk-fed  Your baby receive some breast milk with some feeds by bottle, cup or other ways?  (1 of 4 feeds or less daily; used bottle, finger,cup for any)  Yes – no further questions | | Y = 2 | Y = 2 | Y = 2 | Y = 2 |
| 9) Weaned  Did your baby receive no breast milk?  Yes – no further questions | | Y = 0 | Y = 0 | Y = 0 | Y = 0 |
