# Supplementary material for: Breastfeeding and lactation research: exploring a tool to measure infant feeding patterns
Source: Int Breastfeed J. 2014 Apr 24;9:5. doi: 10.1186/1746-4358-9-5 (PMC4022273; doi:10.1186/1746-4358-9-5)
Supplement: Additional file 3 — Mother’s diary in pamphlet format. [file 1746-4358-9-5-S3.doc]

| **Comments you want to share**  **with the researcher...** |
| --- |

If You Have Any Questions

Please contact

Joy Noel-Weiss at

613-562-5800 x7669 or

Joy.Noel-Weiss@uottawa.ca


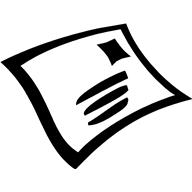


Please return

completed diaries to:

Joy Noel-Weiss

uOttawa School of Nursing

451 Smyth RGN3249C

Ottawa ON Canada K1H 8M5

Participant's ID # _______

**Mother's Diary**

**of Feedings**

| **Week 1** – Please tick your answers at the end of  Week 1 | | |
| --- | --- | --- |
| **For this past week...** | Yes | No |
| I breastfed my baby (any amount) |  |  |
| My baby was only nursed at my breasts |  |  |
| My baby was only nursed at my breasts and I used a tube system (Lactaid, SNS) |  |  |
| My baby was fed by bottle or other way (e.g., finger feeding or cup feeding) |  |  |
| My baby was fed pumped breast milk |  |  |
| My baby was fed other liquids  (e.g., formula or water) |  |  |
| My baby was fed donated breast milk |  |  |
| My baby was fed solids |  |  |

| **Week 4** – Please tick your answers at the end of  Week 4 | | | |  |
| --- | --- | --- | --- | --- |
| **For this past week...** | | Yes | No |  |
| I breastfed my baby (any amount) | |  |  |  |
| My baby was only nursed at my breasts | |  |  |  |
| My baby was only nursed at my breasts and I used a tube system (Lactaid, SNS) | |  |  |  |
| My baby was fed by bottle or other way (e.g., finger feeding or cup feeding) | |  |  |  |
| My baby was fed pumped breast milk | |  |  |  |
| My baby was fed other liquids  (e.g., formula or water) | |  |  |  |
| My baby was fed donated breast milk | |  |  |  |
| My baby was fed solids | |  |  |  |
|  | **Week 2** – Please tick your answers at the end of  Week 2 | | | |
|  | **For this past week...** | Yes | No | |
|  | I breastfed my baby (any amount) |  |  | |
|  | My baby was only nursed at my breasts |  |  | |
|  | My baby was only nursed at my breasts and I used a tube system (Lactaid, SNS) |  |  | |
|  | My baby was fed by bottle or other way (e.g., finger feeding or cup feeding) |  |  | |
|  | My baby was fed pumped breast milk |  |  | |
|  | My baby was fed other liquids  (e.g., formula or water) |  |  | |
|  | My baby was fed donated breast milk |  |  | |
|  | My baby was fed solids |  |  | |

| **Week 5** – Please tick your answers at the end of  Week 5 | | | | |
| --- | --- | --- | --- | --- |
| **For this past week...** | Yes | | No | |
| I breastfed my baby (any amount) |  | |  | |
| My baby was only nursed at my breasts |  | |  | |
| My baby was only nursed at my breasts and I used a tube system (Lactaid, SNS) |  | |  | |
| My baby was fed by bottle or other way (e.g., finger feeding or cup feeding) |  | |  | |
| My baby was fed pumped breast milk |  | |  | |
| My baby was fed other liquids  (e.g., formula or water) |  | |  | |
| My baby was fed donated breast milk |  | |  | |
| My baby was fed solids |  | |  | |
| **Week 3** – Please tick your answers at the end of  Week 3 | | | | |
| **For this past week...** | | Yes | | No |
| I breastfed my baby (any amount) | |  | |  |
| My baby was only nursed at my breasts | |  | |  |
| My baby was only nursed at my breasts and I used a tube system (Lactaid, SNS) | |  | |  |
| My baby was fed by bottle or other way (e.g., finger feeding or cup feeding) | |  | |  |
| My baby was fed pumped breast milk | |  | |  |
| My baby was fed other liquids  (e.g., formula or water) | |  | |  |
| My baby was fed donated breast milk | |  | |  |
| My baby was fed solids | |  | |  |

| **Week 6** – Please tick your answers at the end of  Week 6 | | |
| --- | --- | --- |
| **For this past week...** | Yes | No |
| I breastfed my baby (any amount) |  |  |
| My baby was only nursed at my breasts |  |  |
| My baby was only nursed at my breasts and I used a tube system (Lactaid, SNS) |  |  |
| My baby was fed by bottle or other way (e.g., finger feeding or cup feeding) |  |  |
| My baby was fed pumped breast milk |  |  |
| My baby was fed other liquids  (e.g., formula or water) |  |  |
| My baby was fed donated breast milk |  |  |
| My baby was fed solids |  |  |
