# Supplementary material for: Breastfeeding and lactation research: exploring a tool to measure infant feeding patterns
Source: Int Breastfeed J. 2014 Apr 24;9:5. doi: 10.1186/1746-4358-9-5 (PMC4022273; doi:10.1186/1746-4358-9-5)
Supplement: Additional file 4 — Infant feeding category assessment tool (FeedCat Tool). [file 1746-4358-9-5-S4.doc]

**Additional file 4 - Infant feeding category assessment tool (FeedCat Tool)**

To use the FeedCat tool:

1. Complete Part I by asking a mother the questions.
2. After interviewing the mother, determine feeding category/categories on Part II chart.

**Part I** – Interview questions (using 7 days as the example)

Interviewer: Thank you for participating in the study. I have a few questions about what and how your baby has been fed.

1. What has your baby been fed in the past 7 days (or other option - e.g., 24 hours)?

(prompts - only breast milk? formula? solids?)

1. In the past 7 days, how many times does your baby feed in a day?

How many feeds each day would be breast milk?

If you top up your baby, how much would your baby take in a day?

1. How has your baby been fed past 7 days? (prompts - at breast, by bottle?)
2. Have you used any equipment for breastfeeding? (prompts - nipple shields or nursing supplementers?)
3. Have you used a breast pump? What do you do with the milk?

(note: if mother has said exclusively breastfed, then this question might bring up occasions when baby has been given bottles of expressed milk)

May asked 1 and 3 in reverse order - e.g., How has your baby been fed? asked first

Some RAs ask all questions as 24 hours then all as 7 days, whereas some RAs ask one question for both recall times before moving to next question

**Part II** – After interviewing a mother, complete the following chart to determine a category for type and amount of infant feeding. ONLY circle one Yes (Y) for each timepoint.

| Category  Clarification | Time Points  Age asked &  Time counted | At 1 month,  Past 24 hours | At 1 month,  Past 7 days | At 3 month,  Past 24 hours | At 3 month,  Past 7 days | At 5 month,  Past 24 hours | At 5 month,  Past 7 days |
| --- | --- | --- | --- | --- | --- | --- | --- |
| 1) Exclusively breastfed  Baby received only his mother's milk at her breasts | | Yes | Yes | Yes | Yes | Yes | Yes |
| 2) Exclusively breast milk-fed  Baby received only breast milk (mother or donor) but had **alternate feeding methods**. (e.g., used bottles, cups, spoons, or finger feeds) | | Yes | Yes | Yes | Yes | Yes | Yes |
| 3) Predominately breastfed  Baby received mostly breast milk (75% / 3 of 4 feeds or more) with **all supplements were at his mother's breast** (e.g., used formula with tube at breast and no bottle, finger, cup, other) | | Yes | Yes | Yes | Yes | Yes | Yes |
| 4) Predominately breast milk-fed  Baby received mostly breast milk (75% / 3 of 4 feeds or more) with **alternate feeding methods** | | Yes | Yes | Yes | Yes | Yes | Yes |
| 5) Partially breastfed  Baby received about half breast milk with **all supplements were at his mother's breast** | | Yes | Yes | Yes | Yes | Yes | Yes |
| 6) Partially breast milk-fed  Baby received about half breast milk with **alternate feeding methods** | | Yes | Yes | Yes | Yes | Yes | Yes |
| 7) Minimally breastfed  Baby received some breast milk (25% / 1 of 4 feeds) with **all supplements were at his mother's breast** | | Yes | Yes | Yes | Yes | Yes | Yes |
| 8) Minimally breast milk-fed  Baby received some breast milk (25% / 1 of 4 feeds) with **alternate feeding methods** | | Yes | Yes | Yes | Yes | Yes | Yes |
| 9) Stopped breastfeeding/Weaned//Never breastfed  Baby received no breast milk | | Yes | Yes | Yes | Yes | Yes | Yes |
